# Improvement of attention deficit/hyperactivity disorder (ADHD) in three adult men during testosterone treatment: a case series 

**DOI:** 10.1186/s13256-022-03651-w

**Published:** 2022-11-18

**Authors:** Ane Rogne, Bjørnar Hassel

**Affiliations:** 1 Department of Neurohabilitation, Oslo University Hospital, Kirkeveien 166, 0450 Oslo, Norway; 2 Institute of Clinical Medicine, University of Oslo, Oslo, Norway

**Keywords:** Attention deficit/hyperactivity disorder, ADHD, Impulsivity, Inattention, Testosterone treatment, Sex hormone-binding globulin, Side effects, Case report

## Abstract

**Background:**

Attention deficit/hyperactivity disorder (ADHD) entails inattention, impulsivity, and restlessness at a disabling level. The pharmacological treatment of ADHD rests on the use of centrally acting stimulants, such as methylphenidate and D-amphetamine. In some patients, these drugs cause side effects that preclude their use.

**Case presentation:**

We present three adult male, Caucasian, ADHD patients (24, 37, and 43 years old) whose ADHD symptoms improved during treatment with testosterone. The first patient experienced loss of libido during treatment with methylphenidate; for this, he was offered a trial of testosterone. Unexpectedly, his ADHD symptoms improved with testosterone treatment, and this effect continued with testosterone as monotherapy. The two other patients, who also had side effects from centrally acting stimulants, received testosterone monotherapy with similar results. The effect has now continued for 4.5–5 years at the same doses: 10–60 mg testosterone/day, administered as a skin gel. Prior to testosterone treatment, the patients had serum levels of testosterone in the low–normal range: 12–16 nmol/L (age-specific reference range: 10.4–32.6 nmol/L). The testosterone/sex hormone-binding globulin ratio was low in two patients (0.32 and 0.34; age-specific reference range: 0.38–1.1), suggesting low free serum levels of testosterone. Serum testosterone levels and testosterone/sex hormone-binding globulin ratios increased with testosterone treatment in all patients, but remained within reference values.

**Conclusion:**

These cases suggest that a moderately reduced serum level of free testosterone may contribute to the ADHD symptoms of some adult male ADHD patients, and that testosterone treatment may be of value for these patients.

## Background

Attention-deficit/hyperactivity disorder (ADHD) entails executive problems in the form of inattention, hyperactivity, and impulsivity [1]. Pharmacologically, the treatment of ADHD rests on the use of centrally acting stimulants, such as methylphenidate and D-amphetamine [2]. Sometimes, these drugs have side effects, for example headache [3], anxiety [4], or a reduction in libido [5] that can make their use difficult. ADHD often continues into adulthood [2].

We describe three adult men with ADHD, who responded well to centrally acting stimulants, but who stopped taking them because of side effects. However, they experienced a reduction in ADHD symptoms while receiving testosterone treatment. Prior to testosterone treatment, the patients had serum levels of testosterone in the low–normal range, but the testosterone/sex hormone-binding globulin (SHBG) ratio was below the age-specific reference values in two patients, suggesting low free serum levels of testosterone. Serum testosterone levels and testosterone/SHBG ratios increased with testosterone treatment in all patients, but remained within reference values. This observation suggests that a moderately reduced serum level of free testosterone may contribute to the ADHD symptoms of some adult male ADHD patients.

## Case presentations

### Case 1

A male, Caucasian student, 24 years old, had had attention problems and hyperactivity from early childhood, fulfilling the diagnostic criteria for ADHD [1]. As an adult, he continued to have attention problems and restlessness, and he had difficulties studying. He suffered from insomnia and winter depressions, which is common in ADHD [6]. After his first visit to our clinic, he began treatment with methylphenidate (capsules with extended release), which was increased to 40 mg per day over 3 months. On this dose, his ability to concentrate on his studies improved markedly. He continued this treatment during one winter and did not experience his usual depressive symptoms. Discontinuing methylphenidate immediately led to reduced attention. However, taking this medication led to a reduction in libido, which was troubling to him and his partner. If he discontinued methylphenidate, libido was restored within 1–2 days.

The patient’s secondary sexual characteristics were normal, as was his prostate on rectal examination. Neurological examination and blood pressure (125/80 mm Hg) were normal. His morning serum level of testosterone, SHBG, luteinizing hormone (LH), and follicle-stimulating hormone (FSH) were within reference values, but his testosterone/SHBG ratio, an index of free (active) serum testosterone [7], was below reference values (Table 1). He had normal blood or serum levels of hemoglobin, glycated hemoglobin (HbA1c), glucose, cholesterol, triglycerides, creatinine, prostate-specific antigen, aspartate aminotransferase, alanine aminotransferase, alkaline phosphatase, γ-glutamyl transferase, and albumin. Analyses were done at Oslo University Hospital with a Cobas platform (F. Hoffman-La Roche Ltd., Basel, Switzerland), except for testosterone, which was analyzed by mass spectrometry.

**Table 1:** Testosterone/SHBG ratios and serum levels of hormones and SHBG before and during testosterone treatment

	Patient 1	Patient 2	Patient 3
Testosterone/SHBG (ref.: 0.38–1.1)	0.32 → 0.48	0.34 → 0.60	0.50 → 0.65
Serum testosterone (ref.: 10.4–32.6 nmol/L)	12 → 15	16 → 21	14 → 24
Serum SHBG (ref.: 13.5–57.4 nmol/L)	37 → 31	47 → 35	28 → 37
LH (ref 1.9–9.7 U/L)	3.2 → 2.7	3.1 → 2.8	3.2 → 0.1
FSH (1.5–10.3 U/L)	3.6 → 3.0	3.8 → 3.9	3.6 → 0.4
Testosterone dose	10 mg/day	60 mg/day	50 mg/day

Three adult men with ADHD underwent treatment with a testosterone skin gel as monotherapy. Arrows indicate changes from pretreatment levels to levels after 4–12 months of treatment. Reference values are for healthy men in their thirties from the Nordic countries [7]. Testosterone doses are mg testosterone applied to the skin in the form of a gel each morning [9]. Please note that although serum levels of testosterone and SHBG are within the reference values, the fact that serum testosterone is low whereas SHBG levels are medium, the ratio between them (the testosterone/SHBG ratio) falls below the reference range in Patients 1 and 2

FSH: follicle-stimulating hormone, LH: luteinizing hormone, SHBG: sex hormone-binding globulin, U/L: units/liter

Assessment of ADHD was done with Conners’ Continuous Performance Test 3rd edition (CPT3) [8]. In this PC-based test, patients are shown letters at various inter-stimulus intervals (1, 2, or 4 seconds) and are instructed to press a key on a keyboard in response to each letter, except when the letter “X” appears. CPT3 software provides results as “no indication,” “some indication,” or “strong indication” of dysfunction of attention, impulsivity, sustained attention, and vigilance. The CPT3 scoring software corrects for repeated assessments [8]. In this patient, CPT3 yielded “strong indication of impulsivity” and “some indication of inattentiveness.” There was “no indication” of problems with sustained attention or vigilance.

To improve his lack of sexual interest during methylphenidate treatment, the patient received testosterone skin gel (Testogel, Besins Healthcare, UK) to apply to his upper arms each morning. This decision was supported by his low testosterone/SHBG ratio (Table 1). The dose was 50 mg, as recommended [9]. On this dose, the patient’s sexual interest normalized. His attention, restlessness, and sleep improved in excess of what he experienced during methylphenidate monotherapy. He therefore stopped taking methylphenidate, but the improvement in attention, restlessness, and sleep continued with testosterone as monotherapy. He also felt increased motivation for studies and exercise. After a few months, he changed testosterone treatment to Tostran (Kyowa Kirin Ltd, Galashiels, UK) and reduced the dose gradually to 10 mg/day to reduce side effects in the form of premature ejaculation. (Testogel is sold as sachets, each containing 50 mg testosterone; Tostran is sold as a canister with a piston that delivers doses of 10 mg testosterone per depression).

A dose of 10 mg testosterone/day was sufficient to maintain the improvement in attention, restlessness, and sleep. Similar to what he experienced during methylphenidate treatment, he does not get winter depressions while taking testosterone. The patient still experiences some degree of premature ejaculation, although less so than on the 50 mg/day dose. Discontinuing the testosterone gel ameliorates this side effect after 1–2 days, but his inattention and restlessness then return. Resuming testosterone treatment alleviates ADHD symptoms after 1–2 days of treatment. He has experienced these changes to symptoms and side effects upon pausing or resuming testosterone treatment on several occasions. He remains normotensive and does not experience weight gain, mood disorders, impatience, or aggressiveness. He has now continued testosterone monotherapy for 5 years. He has not received other types of ADHD therapy, cognitive, behavioral, or medical.

Assessment of attention with CPT3 after 4 months of testosterone treatment (10 mg/day) showed improvement of impulsivity (from “strong” to “no” indication of impulsivity) and inattentiveness (from “some” to “no” indication of inattentiveness).

On the initial dose of 50 mg testosterone/day, the patient’s morning serum testosterone level (2–3 hours after administration of the testosterone gel) increased to 28 nmol/L and his SHBG level increased to 40 nmol/L, giving an increased testosterone/SHBG ratio of 0.7. On the maintenance dose of 10 mg testosterone/day, his serum testosterone level remains higher than prior to treatment (Table 1). His testosterone/SHBG ratio remains elevated, in part because his SHBG level is reduced. Serum levels of LH and FSH remain within reference values. His other blood and serum values are normal.

### Case 2

A 37-year-old Caucasian man with a university Master's degree had, since early childhood, suffered from inattention, physical and mental restlessness, impulsivity, and sleep problems. He fulfilled the diagnostic criteria for ADHD [1]. The patient first received methylphenidate at the age of 30 years and experienced improvement of attention, restlessness, and sleep. However, when the effect wore off he felt depressed and angry. He therefore tried D-amphetamine, which also improved symptoms, but caused overactive bladder and left him feeling exceedingly restless when the effect wore off.

The patient’s secondary sexual characteristics were normal, as was his prostate on rectal examination. He was normotensive, and his neurological examination was normal. His morning serum levels of testosterone, SHBG, LH, and FSH were within reference values, but his testosterone/SHBG ratio was below the reference value (Table 1). His other blood and serum values (detailed under Case 1) were normal. Assessment of attention with CPT3 showed “strong indication” of impulsivity, “some indication” of inattentiveness and problems with sustained attention, and “no indication” of vigilance problems.

Because methylphenidate and D-amphetamine had side effects, because his testosterone/SHBG ratio was low, and because testosterone appeared to have an effect on ADHD symptoms in Case 1, the patient was offered a trial of testosterone treatment and started on a dose of 60 mg/day (Tostran), as recommended [9]. On this dose, the patient reported normalization of attention and sleep, while restlessness and impulsivity were much reduced. This effect was noticeable within a week after commencing testosterone treatment. His ability to initiate work remained problematic. He has now continued testosterone treatment for 4.5 years. On two occasions, he has temporarily reduced the dose of testosterone, which has caused the return of ADHD symptoms. He has not received other types of ADHD therapy.

Assessment of attention using CPT3 after 4 months of testosterone treatment yielded scores of “no indication” of problems with impulsivity, inattention, or sustained attention. The patient does not report side effects from the testosterone treatment, for example, weight gain, impatience, or aggressiveness.

After 1 month of treatment with testosterone at 60 mg/day, the patient’s morning serum testosterone level had increased to 40 nmol/L, whereas SHBG was reduced to 36 nmol/L, giving a testosterone/SHBG ratio of 1.1. LH was reduced at 1.5 U/L, whereas FSH remained at 3.0 U/L. After 1 year of treatment, his serum testosterone level and testosterone/SHBG ratio had decreased somewhat, but they remained higher than prior to testosterone treatment (Table 1). LH and FSH were normal, as were his other blood or serum values (detailed under Case 1).

### Case 3

A 43-year-old Caucasian man with full-time employment, married and with two children, had had attention problems, impulsivity, restlessness, and hyperactivity from early childhood, fulfilling the diagnostic criteria for ADHD [1]. He suffered from insomnia and had recurrent winter depressions. The condition was evident in several of his blood relations. As a teenager, he developed motor and vocal tics and was diagnosed with Tourette syndrome. He had tried methylphenidate, which improved attention but caused headache. The patient had previously been a body builder and had experienced relief from his attention problems and restlessness when he used testosterone at high doses as an anabolic steroid. He had stopped using testosterone 10 years earlier because it led to gynecomastia and liver cysts.

On examination, the patient had normal secondary sexual characteristics. His blood pressure was 150/100 mm Hg. Rectal examination of the prostate was normal. His neurological examination was notable for tic-like facial movements and eye closure, but was otherwise normal. Gynecomastia was no longer present. His morning serum testosterone level was within reference values (Table 1), as were SHBG, LH, FSH, and the testosterone/SHBG ratio. His other blood and serum values (detailed under Case 1) were normal. The patient started testosterone treatment before assessment of attention with CPT3 was possible and did not wish to stop treatment for the sake of CPT3 testing.

Because of the patient’s hypertension, he first started treatment with bendroflumethiazide, which after 1 month had lowered his blood pressure to 125/75 mm Hg, but had not improved attention or restlessness. Because of the previous adverse effect of methylphenidate and his earlier experience that testosterone improved ADHD symptoms, the patient was offered a trial with testosterone gel, 50 mg/day (Testogel), as recommended [9].

Within 1 week of testosterone treatment, the patient experienced improvement of attention and restlessness. Over the next few months, he also experienced a reduction in seasonal depressive symptoms and in sleep problems. He reports side effects in the form of reduced volume of ejaculates. His Tourette syndrome symptoms (vocal and motor tics) have not changed during testosterone therapy. He does not experience psychological side effects, such as irritability or aggression. His blood pressure is 130/90 mm Hg. He has now continued testosterone treatment as monotherapy at the same dose for 5 years. He does not receive other kinds of ADHD therapy, cognitive, behavioral, or medical.

After 6 months of testosterone treatment, the patient’s serum testosterone level 2–3 hours after administration of the testosterone gel had increased, as had his testosterone/SHBG ratio (Table 1). LH and FSH levels were much decreased, indicating pituitary suppression and (reversible) reduction of sperm production [10], which was not a concern according to the patient. His other blood and serum values (detailed under Case 1) remain normal.

## Discussion

The three patients described here reported improvement of their ADHD symptoms during testosterone treatment, although all three patients had normal secondary sexual characteristics and serum testosterone levels that, although low, were within reference values. Thus, the patients were not hypogonadal. However, in two of our patients, the testosterone/SHBG ratio was below the reference value, an indication that the serum level of free (active) testosterone was low [7]. The testosterone/SHBG ratio increased to normal values with testosterone treatment, suggesting that an increase in free testosterone levels was involved in the improvement of ADHD symptoms. A predominant placebo effect was not highly likely, since all patients chose to continue treatment for several years. The close temporal relationship between commencing, or resuming, testosterone treatment and amelioration of ADHD symptoms in two patients supports the inference that testosterone was causally involved in the improvement of ADHD symptoms. Improvement of seasonal (winter) depressions, which is common in ADHD [6] may have contributed to the overall clinical effect of testosterone therapy; however, ADHD symptoms improved even during non-depressive (summer) periods, suggesting an effect on ADHD symptoms per se. It may be speculated that the effect of testosterone on ADHD symptoms in part was owing to the improvement of sleep. Sleep problems and fatigue are common in ADHD patients [11]. While an effect of testosterone on sleep and fatigue was beneficial to our patients, the results of CPT3 testing were rather ADHD-specific, pointing to a genuine effect of testosterone on attention and impulsivity.

In an experimental study on Macaca monkeys, long-term methylphenidate treatment led to a reduction in serum testosterone [12]. Case reports suggest a similar effect in humans [13, 14]. In the first patient, such an effect on testosterone levels may have contributed to the loss of libido during methylphenidate treatment, and testosterone treatment may have corrected this. In agreement with previous studies [15, 16], SHBG levels decreased with testosterone treatment in two patients, an effect that contributed to increasing their testosterone/SHBG ratio.

At present, we cannot precisely delineate the mechanism of action behind testosterone’s improvement of ADHD symptoms in our patients. Testosterone modulates brain function through activation of neuronal androgen receptors, which triggers changes in gene transcription [17]. Studies in humans and animals have shown that areas that are important for cognitive function, for example, the hippocampus, amygdala, and prefrontal cortex, are rich in androgen receptors and that manipulation of androgen receptors affects executive function [17–19]. This could be why testosterone treatment improved ADHD symptoms in the presented patients, who all suffered from problems with executive function, such as inattention and impulsivity. Testosterone also modulates plasma membrane receptors for classical neurotransmitters such as monoamines, glutamate, and gamma-aminobutyric acid [20] and so may improve cognition through several cellular mechanisms. Further, in the brain, aromatase may convert testosterone into estradiol, which mediates some of the cognitive effects of testosterone by activation of estrogen receptors; this applies to both men and women [21]. An interaction between the dopaminergic system, which has a special role in ADHD [22], and estrogen could be important, especially in women with ADHD [23].

The doses used in the present report are the same as those used in men with hypogonadism or aging-related low serum levels of testosterone [24, 25]. The difference between our patients with respect to the effective dose of testosterone may have been due both to pharmacokinetics and pharmacodynamics. From Table 1 it can be seen that there is no linear relationship between testosterone dose and increase in serum levels of testosterone, suggesting differences between patients with respect to transdermal absorption or the metabolism of testosterone, which is in line with a previous study [26].

Side effects from testosterone were tolerable to the patients and did not cause them to stop treatment. Of special importance was the absence of aggressiveness; increased aggressiveness could have been deleterious in persons with a high degree of impulsivity. This observation agrees with a recent study on testosterone therapy in traumatic brain injury patients [27]. We monitored patients closely with respect to plasma lipids, prostate-specific antigen, hemoglobin, and other blood and serum markers and found no sign of adverse effects of the testosterone treatment at the doses used. Follow-up of the patients was too short for a comprehensive evaluation of long-term health effects, but testosterone therapy is considered safe with respect to development of cardiovascular disease and prostate cancer [28, 29]. Nevertheless, patients on testosterone therapy should probably be monitored regularly with respect to polycythemia and plasma lipids, liver, prostate, and cardiac function, blood pressure, sleep apnea, irritability, and mood changes [30–32].

### Strengths and limitations

A strength of this report is that the patients’ use of the testosterone gel was mirrored in their serum levels of testosterone, which documented adherence to the treatment. Further, the subjective improvement of ADHD symptoms was corroborated in the two patients who underwent CPT3 testing before and after commencing testosterone treatment. A limitation of this report is the fact that we did not expose the patients to a placebo formulation of the testosterone gel. However, two patients temporarily stopped treatment or reduced the dose of testosterone and experienced return of ADHD symptoms, which confirmed an effect of testosterone on their ADHD symptoms. Another limitation is the lack of background knowledge of the importance of testosterone for normal cognitive function in adult men in their daily lives. Most studies of testosterone and cognitive function are done in ageing men [33] or in men who receive anti-testosterone treatment as part of prostate cancer therapy [34], or they are laboratory investigations with the use of single high doses of testosterone [35], which probably have modest relevance for the understanding of the cognitive effects of long-term testosterone treatment of younger men at moderate doses.

## Conclusion

Our observation suggests that a moderately reduced serum level of free testosterone may contribute to the ADHD symptoms of some adult male ADHD patients, and that testosterone treatment may be of value in the treatment of these patients.

### Patient perspectives

Patient 2

After several years of struggling with undiagnosed ADHD symptoms combined with dealing with the effects of other severe health issues comprising arthritis, brain surgery, and sclerosis, one had but given up when the ADHD was diagnosed and racemic drug treatment was started. However, the negative side effects of drug treatment, for example heightened nervousness and stress and an overactive bladder, proved difficult and straining on one’s psyche and overall energy levels, which regardless of the positive effects the drugs had on inattention, restlessness, and sleep, rendered the effect of the treatment somewhat wanting. Upon starting hormone treatment, to one’s surprise, the perceived positive effects were equal to that of drug treatment. Furthermore, the treatment has seemed to vanquish symptoms stemming from other health issues. The hormone treatment has left one feeling more calm, grounded, solid, and sound and, for the first time, able to relax and actually “charge the batteries.” Especially the alleviation, or rather abolishment, of one’s constant fatigue is hard to describe in words. In conclusion, one has never felt more functioning and healthy. It has more or less felt like “magic.”

Patient 3

I am one of the patients described in this publication, a man in my mid-forties who, from early childhood, has struggled with attention problems, impulsivity, restlessness, and hyperactivity. From my late teens, I also have suffered from recurrent depressions each winter. I got the ADHD diagnosis at the age of 7 years and Tourette syndrome in my early teens. I have received treatment with testosterone skin gel, 50 mg/day for the last 4.5 years, and have experienced a marked reduction in my ADHD symptoms, experiencing improvement especially in attention, physical, and mental restlessness, and thereby sleep. One of the most positive effects for me is that I have not experienced the usual depressive symptoms since I started this treatment.

## Data Availability

The data that support the findings of this study are available from the corresponding author, but restrictions apply to the availability of these data, which were used under license for the current study, and so are not publicly available. Data are however available from the authors upon reasonable request and with permission from the patients described in this report.
